# Changes in Texture and Collagen Properties of Pork Skin during Salt–Enzyme–Alkali Tenderization Treatment

**DOI:** 10.3390/foods13203264

**Published:** 2024-10-14

**Authors:** Qiang Zou, Yuyou Chen, Yudie Liu, Linghui Luo, Yuhan Zheng, Guilian Ran, Dayu Liu

**Affiliations:** 1 School of Food and Biological Engineering, Chengdu University, Chengdu 610106, China; zouqiang119@126.com (Q.Z.); chenyuyou@stu.cdu.edu.cn (Y.C.); liuyudie@stu.cdu.edu.cn (Y.L.); luolinghui@stu.cdu.edu.cn (L.L.); zhengyuhan@stu.cdu.edu.cn (Y.Z.); ranguilian@stu.cdu.edu.cn (G.R.); 2 Meat Processing Key Laboratory of Sichuan Province, School of Food and Biological Engineering, Chengdu University, Chengdu 610106, China

**Keywords:** salt–enzyme–alkali staged tenderization, secondary structure, pork skin, textural characteristics, collagen

## Abstract

The effects of salt–enzyme–alkali progressive tenderization treatments on porcine cortical conformation and collagen properties were investigated, and their effectiveness and mechanisms were analyzed. The tenderization treatment comprised three treatment stages: CaCl_2_ (25 °C/0–30 min), papain (35 °C/30–78 min), and Na_2_CO_3_ (25 °C/78–120 min). The textural, microscopic, and collagenous properties (content, solubility, and structure) of pork skin were determined at the 0th, 30th, 60th, 90th, and 120th min of the treatment process. The results showed that the shear force, hardness, and chewability of the skin decreased significantly (*p* < 0.05), and the elasticity exhibited a gradual increase with the progression of tenderization. The content and solubility of collagen showed no significant change at the CaCl_2_ treatment stage. However, the soluble collagen content increased, the insoluble collagen content decreased, and the collagen solubility increased by 18.04% during the subsequent treatment with papain and Na_2_CO_3_. Meanwhile, the scanning electron microscopy results revealed that the regular, wavy structure of the pig skin collagen fibers gradually disappeared during the CaCl_2_ treatment stage, the overall structure revealed expansion, and the surface microscopic pores gradually increased during the papain and Na_2_CO_3_ treatment stages. The findings of the Fourier transform infrared spectroscopy analysis indicated that the hydrogen bonding interactions between the collagen molecules and the C=O, N-H and C-N bonds in the subunit structure of collagen were substantially altered during treatment and that the breakage of amino acid chains and reduction in structural ordering became more pronounced with prolonged treatment. In the tertiary structure, the maximum emission wavelength was blue-shifted and then red-shifted, and the fluorescence intensity was gradually weakened. The surface hydrophobicity was slowly increased. The salt–enzyme–alkali tenderization treatment considerably improved the physical properties and texture of edible pork skins by dissolving collagen fibers and destroying the structure of collagen and its interaction force.

## 1. Introduction

Pork skin is a by-product of animal husbandry, and it is widely used in some countries and regions in Asia, Europe, and South America. In particular, China is a major hog-farming country that produces a large amount of pork skin by-products in slaughtering and processing every year, which account for approximately 10% of the total weight of the pig carcass. In the current context of global sustainable development, the food production sector faces major challenges, such as resource scarcity and environmental pollution. Utilizing pig skin by-products not only helps to reduce waste in the food industry, but also significantly improves the efficiency of resource utilization, thus providing new solutions for sustainable food production. Pork skin has unique functions in human production and life, and it is important to extract high-quality bioactive peptides or collagen from pig skin to increase its added value. Bioengineered corneas based on pig skin collagen helped 14 blind patients to regain their sight, according to Rafat et al. [1]. In addition, pig skin has shown unique advantages in the production of cosmetics. Meanwhile, pork rinds contain abundant amounts of collagen; minerals, such as calcium, phosphorus, magnesium, and iron; and various vitamins. They have a unique chewy and elastic texture and are thus processed into popular ready-to-eat food products, such as soy sauce pork rinds and pickled pepper pork rinds in the Chinese market. However, the collagen in pork rinds exists in the form of collagen fibers, whose triple-stranded helical structures and intermolecular cross-linking result in poor tenderness and toughness after heating, which affect consumer acceptance [2]. Meat tenderness is the most important factor influencing customer preferences and is usually assessed to determine the quality of the meat [3]. Although pig skin is mainly used for leather processing, a small amount is currently considered a food ingredient. The utilization rate of pig skin remains at 40%. To expand the development and consumption of pig skin products in the market, they are tenderized to improve the product quality and market acceptance. Meat tenderness is subject to the influence of many biological and environmental factors, such as the collagen content [4], item breed and age, calpain and calpain inhibitor proteins, and preslaughter and post-slaughter treatments [5]. The currently available main physical tenderization methods include ultra-high-pressure, shock-wave, ultrasonic, and resonance techniques. However, these techniques suffer from an insufficient tenderizing effect, high equipment costs, and an inability to adapt to mass production. Therefore, in consideration of the economic benefits, chemical tenderization methods and biological tenderization methods remain the preferred methods for the tenderization of meat in Chinese enterprises. The most commonly used chemical tenderizers comprise phosphate, alkali, organic acid, and so on [6]. These chemical tenderizers effectively improve the tenderness and quality of pork skin. The tenderization mechanism of salt is mainly achieved by improving the water retention of meat, reducing the thermal stability of meat collagen, and enhancing the activation of calcium-activated enzymes [7]. Koohmaraie et al. [6,8] demonstrated that the injection of CaCl_2_ either into the carcass or directly into the muscle-activated calpain improved the meat tenderness after bloodletting. Plant proteases are used to hydrolyze collagen, elastin, and muscle proteins to improve meat tenderness. Papain exhibits high stability and a strong hydrolytic capacity for proteins, peptides, esters, and other compounds [9]; destroys myofibrillar proteins and connective tissue in meat [10]; and accelerates protein hydrolysis, which results in considerable improvements in tenderness and flavor [11]. Biotenderization achieves its tenderizing effect through the degradation of connective tissue and muscle proteins via the actions of exogenous proteases, which reduce the shear force of the meat. Alkalinity raises the environmental pH, which in turn induces collagen dissolution in muscle and connective tissue, which results in softer meat [12]. Sheard and Tali [13] added an alkaline marinade to pork tenderloin, which increased the pork pH from 7.22 to 8.93 and reduced the shear force from 5.1 N to 2.9 N. Depending on the various tenderizing mechanisms of salts, enzymes, and alkalis, the introduction of moisture and the alteration of the pig skin structure using calcium salts render it loose and hygroscopic. On this basis, enzyme treatment further breaks down the collagen in pork skin to promote its solubilization and hydrolysis, and alkaline conditioning aids in changing the pH of pork skin to further promote protein solubilization and decomposition. Preliminary research reports on Chinese meat enterprises show that the salt–enzyme–alkaline staged tenderization treatment is currently gaining acceptance and being used in practical applications in the industry [12,14]. However, no study has reported the use of the salt–enzyme–alkali staged tenderization of pork skin and the relationship between the collagen properties and texture of the pork skin after such treatment.

Collagen is the major structural protein in the extracellular matrix and comprises one or more alpha chains, forming a triple-helix structure. These collagen molecules aggregate in the extracellular matrix to form supramolecular structures [15]. Collagen contributes to the formation of highly diverse connective tissue types, such as skin, tendons, and cartilage, through a complex series of biosynthetic processes, including intracellular and extracellular posttranslational modifications, supramolecular assembly, and natural cross-linking [16]. Previous studies have established a relationship between the connective tissue characteristics (quantitative, chemical, and structural information of collagen) and the texture of meat and meat products. The total collagen content showed a positive correlation with the texture and tenderness of raw collagenized meat and beef, respectively [17]. As animals age, unstable divalent bonds gradually mature and evolve into trivalent ones under the action of reactive oxygen species, etc., with notable increases in shear, hardness, and chewiness. The regulation of pork skin tenderness using salt, plant proteases, and alkali is associated with the intramolecular denaturation of collagen and the contraction of collagen fibers [18]. Salt and alkalis directly regulate the endogenous enzyme activity by increasing the ionic strength and altering the pH while disrupting the collagen fiber structure, and they cause a corresponding decrease in the insoluble collagen content [19]. In addition, papain can trigger the breakdown of the long-chain collagen structure into short-chain peptides and amino acids, which reduces the fiber bundle structure and cross-linking points of collagen. However, the relationship between collagen characterization and meat products requires further investigation. The effect of collagen characterization on texture development in collagen-based meat products, especially during the tenderization process, has not been fully discussed. Therefore, this study aimed to investigate the effects of the salt–enzyme–alkali staged tenderization treatment method on the texture and collagen properties of pork skins, establish the relationship between the texture of pork skins and the collagen properties of connective tissue, and investigate the changes in the structure and properties of pork skin collagen during the treatment. It aimed to provide effective technical support for the quality improvement of pork skin products and related collagen-based meat products. It is also recommended to explore the effects of other enzymatic or chemical treatments on the collagen properties to assess their potential in improving the texture of pig skin.

## 2. Materials and Methods

### 2.1. Materials

The pig skins used in this experiment, which were purchased and stored in a −20 °C environment, were all leg skins from 1-year-old reared ground hogs with the same feeding environment and feed formulation, with an average weight of about 190–220 kg, from the Longquan Breeding Base, Chengdu City, Sichuan Province, China.

### 2.2. Tenderization of Pig Skin

The pork skin was thawed in a constant-temperature and -humidity oven (KK-150 type; Shanghai Keken Test Equipment Co., Ltd., Shanghai, China) at 4 °C for 6 h. The surface fat and stray hairs of the pork skin were removed, and the skin was cleaned and then cut into pieces with a mass of about 20 g. Then, the skin was processed in a 0.67% CaCl_2_ solution at 25 °C for 30 min, followed by a 340 U/g papain solution at 35 °C for 48 min. The pretreated pork skin was placed in a 25 °C, 0.67% CaCl_2_ solution for 30 min, followed by a 35 °C, 340 U/g papain solution for 48 min, and finally in a 25 °C, 0.24% food-grade Na_2_CO_3_ solution for 42 min. The salt–enzyme–alkali tenderization had a total treatment time of 120 min and a material ratio of 1:5. The treatment was performed after constant-temperature immersion in a digital-display thermostatic water bath (S-CH-4A type; Shanghai Sheyan Instrument Co., Ltd., Shanghai, China). (The concentrations of salt, enzyme, and alkali and the treatment times were derived from the response surface results from the previous pre-tests). Sampling points were obtained at 0, 30, 60, 90, and 120 min during the salt–enzyme–alkali tenderization process.

### 2.3. Proximate Analysis

Proximate analyses were performed on pork skins sampled at various tenderization times. The moisture, protein, and fat content was determined in accordance with the Chinese National Standards (GB5009.3-2016 [20], GB5009.5-2016 [21], and GB5009.6-2016 [22]), respectively.

### 2.4. Determination of Shear Force

Approximately 20 g pig skin was collected, and the samples were measured using a texture analyzer (TA-XT Plus type, Stable Micro Systems, Godalming, Surrey, UK) with a sample thickness of 1 cm. The HDP/BSW shear probe was applied in the measurement with the following conditions: pre-testing rate of 2.0 mm/s, testing rate of 2.0 mm/s, and post-testing rate of 10.0 mm/s; target mode selection of strain 50%, trigger force of 15.0 g, and duration of 5 s [18,23]. All tests were repeated thrice for the averaging of the results.

### 2.5. Texture Profile Analysis

The TPA method was used to evaluate the sample compression-based textural properties using a texture analyzer (Model, TA. XT; Make, Stable Microsystems, Godalming, Surrey, UK) equipped with a 1 kg piezometric element and a 20 mm cylindrical probe (P/20). All samples were cut into pieces measuring 1 cm in thickness, 3 cm in length, and 3 cm in width. The test parameters were set as follows: pre-test speed of 2 mm/s, during-test speed of 1 mm/s, post-test speed of 2 mm/s, trigger force of 10 g, sample height of 10 mm, measurement interval of 5 s, and test compression ratio of 50%. The hardness, chewability, and elasticity were determined by the method of Bourne [24] and calculated using the User’s Guide software version 23.0.

### 2.6. Scanning Electron Microscopy (SEM)

To observe the changes in the collagen fiber network structure of the pig skin tissue, we performed modifications in accordance with the method of Song et al. [25]. The pork skins were cut to lengths and widths of 0.5 cm × 0.5 cm and immersed in 10% sodium hydroxide solution for 3 days at room temperature. After rinsing with distilled water for 10 h, the samples were fixed in a phosphate buffer (0.1 mol/L, pH 7.2) containing 15 mL 2.5% glutaraldehyde (*v*/*v*) at 4 °C for 24 h. The fixed samples were washed thrice with phosphate buffer, with each wash lasting 15 min, and then dehydrated with 30%, 50%, 70%, 90%, and 100% ethanol solutions for 1 h each time. The samples were dried in a vacuum freeze dryer (FD-12B-50T type, Shanghai Sheyan Instrument Co., Ltd., Shanghai, China) for 24 h. The dried samples were sprayed and coated with gold via SEM (EDAX SX-40, AKASHI, Sakatao City, Japan) at an accelerating voltage of 20 kV to observe the pig skin microstructure.

### 2.7. Collagen Characteristics (Content and Solubility)

The soluble and insoluble collagen content of the pig skin samples was determined by following the method of Wattanachant et al. [26], with slight modifications. Five sample groups were weighed into a 2 g solution (0.5 mM CaCl_2_, 1.5 mM KCl, and 32.8 mM NaCl) homogenized at 10,000 rpm for 2 min. Centrifugation was performed at 25 °C for 25 min at 10,000 rpm, and the extraction and centrifugation steps were repeated twice to obtain the supernatant. After the above operations, the soluble and insoluble collagen content was determined through hydrolysis with 30 mL 3 M sulfuric acid at 105 °C for 16 h. The hydroxyproline content of the hydrolysate was analyzed following the Chinese national standard (GB/T9695.23-2008) [27] and converted to the collagen content using a factor of 7.25 [28]. The total collagen content was expressed as the sum of the soluble and insoluble collagen content, and the collagen solubility was expressed as the quotient of the soluble and total collagen content.

### 2.8. Extraction of Collagen from Pig Skin

Porcine skin collagen was extracted as described in the work of Senaratne et al. [29], with slight modifications. Five sets of samples were cut into small pieces. The samples were rinsed with 10% NaCl solution for 2 h to remove impurities. Next, the pork skins were soaked in 0.1 mol/L NaOH solution for 48 h to remove pigments and impurity proteins, followed by soaking in 10% n-butanol solution for 24 h to remove excess fat. The samples were washed to a neutral pH with purified water, combined with 0.1 mol/L acetic acid solution containing 1% pepsin at the solid–liquid ratio of 1:10, and stirred for 72 h. The filtrate was collected and salted out, centrifuged, dialyzed, and vacuum freeze-dried to obtain collagen, and the samples were placed in a refrigerator at −20 °C to determine the indices. All of the above operations were performed at a low temperature.

### 2.9. Fluorescence Spectrum

A certain amount of freeze-dried pig skin was collected, and 0.5 mg/mL collagen solution was prepared through the dissolution of the collagen of samples with 0.1 mol/L glacial acetic acid solution. Five sets of pig skin collagen fluorescence spectra (400–300 nm) were scanned using a fluorescence spectrum analyzer (SYNERGYH1MG, PerkinElmer, Wellington, New Zealand). The excitation wavelength was set at 280 nm, and the slit width was 5 nm.

### 2.10. FTIR Spectroscopy

Five sets of samples were analyzed via FTIR spectroscopy in accordance with the method of Noorzai et al. [30], with slight modifications. After freeze drying, a 1 mg pig skin sample was obtained, combined with 100 mg KBr in a mortar, and ground well until no more particles were observed. The ground sample was pressed into a translucent thin slice and scanned using an FTIR spectrometer (Spectrum 3, Agilent Technologies Inc., Santa Clara, CA, USA) at 400–4000 cm^−1^ with a resolution of 4 cm^−1^ and 32 scans.

### 2.11. Surface Hydrophobicity Determination

To determine the surface hydrophobicity of pig skin collagen, we slightly modified the method in accordance with the work of Chin et al. [31]. A certain amount of freeze-dried pig skin collagen was collected and dissolved in 0.1 mol/L glacial acetic acid solution to prepare a 1.0 mol/L pig skin collagen solution under various tenderization treatment times. A total of 1 mL of the above collagen solution and 200 µL 1.0 mg/mL bromophenol blue solution were placed in a centrifuge tube, vortexed for 10 min, and centrifuged at 8000 rpm for 10 min at 4 °C. A total of 0.5 mol supernatant was obtained, combined with 4.5 mol 0.1 mol/L glacial acetic acid solution, and then mixed uniformly. The absorbance values of the collagen solutions of various samples were measured at 595 nm, with 0.1 mol/L glacial acetic acid solution serving as a blank group. The surface hydrophobicity of collagen was calculated as follows:

$$\mathrm{Bromophenol}\;\mathrm{blue}\;\left(\mu g\right)=\frac{A_{\mathrm{blank}}\;-\;A_{\mathrm{sample}}}{A_{\mathrm{blank}}}\times \;200$$

### 2.12. Statistical Analysis

The experimental data were plotted using the IBM SPSS software (version 23.0; SPSS, Chicago, IL, USA) software with Origin 2021. One-way analysis of variance, least significant difference tests, and multiple comparisons of the physicochemical data were performed. Tukey’s significant difference test was conducted to test the significant differences among all statistical analyses at the 5% significance level (*p* < 0.05). Different letters in all tables and figures indicate significant differences at the *p* < 0.05 level. All tests were repeated thrice.

## 3. Results

### 3.1. Proximate Composition of Pig Skin

The effect of the tenderizing process on the nutrient composition of pork rinds was analyzed to further evaluate its food value. Table 1 shows the influence of different tenderization periods on the nutritional composition of pork skin. The moisture content of the pork skin increased gradually during the 0–90 min treatment stage and decreased slightly during the 90–120 min treatment stage. The protein and fat content decreased gradually with the increase in the tenderization treatment period. This outcome is consistent with the findings of Hoffman et al. [32], which proved the presence of a certain negative correlation between the moisture content and fat content in meat. Proteins, as the main components of pork skin, usually show a close relation to the flavor, tenderness, and nutritional value of meat in meat quality evaluations in terms of the protein content and mode of presence. The staged tenderization treatment resulted in significantly reduced protein content in the pork rinds (*p* < 0.05). This difference may be due to the increased moisture content of pork rinds due to the introduction of additional water via the tenderization treatment method or the alteration of their internal moisture distribution; in addition, changes in the proportion of moisture in the total nutrients may indirectly affect that of other nutrients [33].

### 3.2. Change in Shear Force

During the salt–enzyme–alkali tenderization of the pork skin, the pork skin shear force decreased significantly (*p* < 0.05) with the progression of tenderization, as observed in Table 1. The decrease in the shear force of pork skin at a treatment time of 30 min was observed mainly because the Ca^2+^ in CaCl_2_ increased the activity of calcium-activated proteases, which rendered the myofibrillar structural proteins unstable. In addition, CaCl_2_ can promote the dissolution of myosin and actin, which reduces the hardness of pork skin and enhances water retention [34]. Gerelt et al. [35] discovered a considerable improvement in the shear force of pork skin by submerging dehydrated meat in a 150 mM CaCl_2_ solution, which significantly improved the tenderness of the meat, without adversely influencing other taste and quality characteristics. A significant decrease in the shear force was observed at 60 min (*p* < 0.05). This result was attributed to the capability of papain to properly degrade collagen, elastin, and myofibrillar proteins in meat, which results in the disruption of their reticular structures and an increase in protein solubility. Lyng et al. [36] conducted experiments using test tubes, which revealed that protease was active on myofibrils and collagen. At 60–120 min, Na_2_CO_3_ caused an increase in the ambient pH, which can improve electrostatic repulsion and promote myofibrillar fibrin and collagen dissolution. When the ambient pH was increased, it promoted pork skin tenderization. This result is consistent with that of Petracci et al. [35,37], who reported that their samples had higher cooking yields and more tenderized meat when the pH was increased.

### 3.3. Changes in Texture

TPA based on double-cycle compression can simulate the chewing process of pig skin and provide the textural parameters of the samples, such as the hardness, chewiness, and elasticity [38]. Table 1 displays the changes in the TPA parameters of pig skin during the combined tenderization process. The salt–enzyme–alkali combined tenderization treatment significantly affected the textural properties of the pig skin (*p* < 0.05). From 0 min to 30 min, the CaCl_2_ treatment exerted a weak effect on the hardness of the pig skin. At 60 min, the papain treatment was still not finished, but the hardness of the pig skin decreased significantly more than that at 0 min to 30 min and at 60–120 min. At 60–120 min, the pig skin presented a continuous decrease in hardness. In addition, the chewability of the pig skin slowly decreased with prolonged treatment. This result is consistent with the effects of CaCl_2_, enzymes, and alkalis on the texture of meat pieces as reported by Boleman et al. [39] and Bhat et al. [5]. The textural properties of meat products depend mainly on the type and content of structural proteins and their alterations during processing [40]. Dominguez-Hernandez et al. [41] showed that the denaturation and degradation of collagen in the connective tissue may improve the tenderness of meat. Papain and alkaline environments disrupt the triple-helix structure of collagen; in addition, the breakage of hydrogen bonds loosens its molecular chains, and water molecules enter the three alpha chain gaps of collagen. This condition results in an increased diameter and the contraction of the collagen fibers, which often shortens them by one-quarter of their original length [42]. The intermolecular covalent bonds of collagen fibers stabilized during salt–enzyme–alkali tenderization may break during further enzyme and alkali treatments, which leads to the destruction of the collagen fibers. Therefore, the substantial reduction in the firmness and chewability of salt–enzyme–alkali-tenderized pork skins may be related to the decrease in collagen fiber tension caused by collagen degradation and ion exchange.

The elasticity values increased during the tenderization treatment. The papain treatment stage resulted in a lower degree of change in the elasticity value of pork skin than the Na_2_CO_3_ treatment stage. López et al. [43] also showed that papain had an inferior effect on meat elasticity compared with alkali. The improvement in the skin texture in the Na_2_CO_3_ treatment stage was significant because the OH^−^ produced by the dissociation of Na_2_CO_3_ interacted with the amino acids in the pork skin proteins, which caused the breakage of the ionic and hydrogen bonds within the protein molecules; this condition resulted in the relaxation and expansion of the skin structure and an improvement in its textural properties [44]. Similarly, Galato et al. [45] formulated a combination of Na_2_CO_3_, table salt, and tripolyphosphate into a marinade before injecting it into pork blocks; the results showed that the pH of the meat blocks in the marinade group containing Na_2_CO_3_ and tripolyphosphate was increased by an average of 0.3–0.5, and the samples had significantly higher juiciness and a significant increase in the final yield and overall tenderness.

### 3.4. Changes in Pig Skin SEM Observations during Tenderization

**Figure 1:**
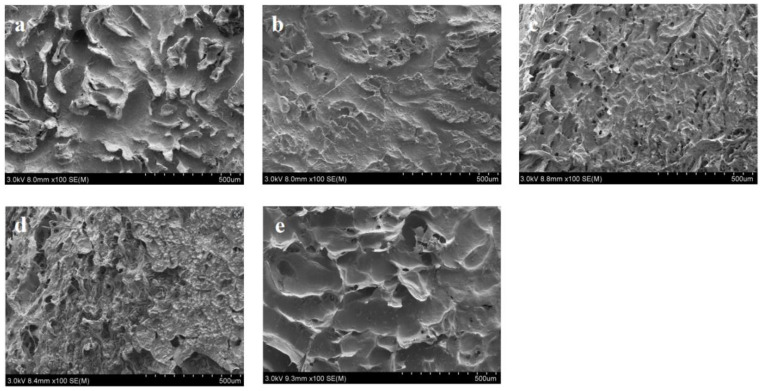
Effect of tenderization process on microstructure of pork skin observed via SEM with selected magnification of 100×. (**a**) Untreated stage (0 min); (**b**) 30, (**c**), 60, (**d**) 90, and (**e**) 120 min stages.

Figure 1 shows the microstructure of the pig skin during the salt–enzyme–alkali tenderization treatment. At 0 min, the collagen fibers were tightly arranged and regular, and the tissue structure was complete and orderly. The tenderization treatment causes the collagen fibers in the pig skin, which have a regular wave structure, to disappear. This treatment significantly reduces the number of collagen fibers, while increasing both the fiber gaps and the fiber diameter. From this result, it can be hypothesized that the increase in the fiber gap and diameter was due to the dissolution of collagen and the entry of water molecules into the collagen triple helix during the combined tenderization process [18]. Compared with that at 0 min, at 30 min of tenderization treatment, the structure of the pig skin changed slightly. The surface exhibited slight flattening, the collagen fibers contracted and deformed slightly, gaps appeared between the fibers, and a small number of holes formed. After 60 min of tenderization treatment with papain, the fibrous tissue of the pork skin was enlarged, the structure became looser, and more microscopic holes appeared on the surface. Qihe et al. [46] found that papain could decompose collagen fibers and myofibrillar fibers in meat, relax the fiber structure, promote water penetration and absorption, enhance the water retention of meat, and increase the water flow in meat. After Na_2_CO_3_ treatment, the pork skin exhibited a rough and irregular surface, the spacing between the collagen fibers increased, and the fibers showed a loose and disordered arrangement. Silva et al. [47] pointed out that the collagen fibers on the meat surface were partially dissolved and structurally altered in a high-pH environment. More complete damage to the skin structure was observed at 120 min of tenderization. The proteolysis on the surfaces of the pork rinds was severe, the pore structure was damaged, and the pore size was enlarged. This result may have been due to the staged progressive treatments with CaCl_2_, papain, and Na_2_CO_3_, promoting the dissolution of the collagen fibers and myofibrils via the regulation of the ionic balance of the pig skin tissue, which led to severe damage to the structural integrity of the pig skin and a better rejuvenation effect. The SEM results were highly consistent with those of TPA. The degradation of collagen fibers may reduce the mechanical strength of the connective tissue [48], and this can be reflected in alterations in collagen solubility.

### 3.5. Changes in Collagen Content and Solubility

**Figure 2:**
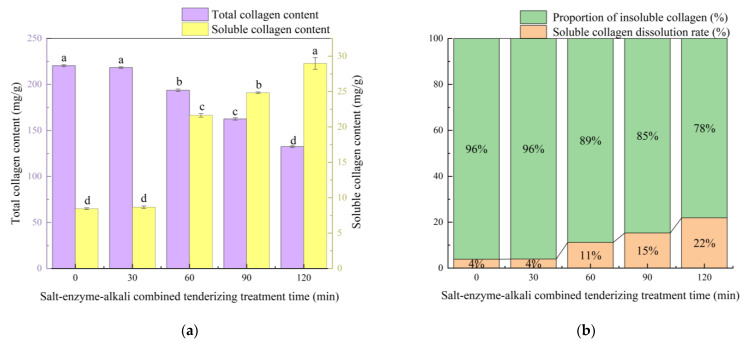
Changes in collagen content and solubility in pig skin during salt–enzyme–alkali combined tender treatment. (**a**) Changes in the total and soluble collagen content of pig skin during tenderization treatment; (**b**) changes in the dissolution rate of pig skin collagen during tenderization treatment. Different letters (a–d) indicate significant differences between different samples (*p* < 0.05).

Torrescano et al. [49] showed that the collagen content and solubility are important factors influencing meat tenderness; the higher the collagen content, the poorer the tenderness of meat products, i.e., collagen and meat tenderness are negatively correlated. The changes in the collagen content and solubility of pork skin during salt–enzyme–alkali tenderization were investigated, and the results are shown in Figure 2. The changes in the total and soluble collagen content showed no significance (*p* > 0.05) during the CaCl_2_ treatment stage. This result is consistent with the findings reported by Morgan et al. [50], who found no differences in the quantitative collagen types (total, thermosoluble, or thermo-insoluble collagen) when mature cows were injected with CaCl_2_ compared with the control muscle. The papain and Na_2_CO_3_ treatment phases resulted in a significant (*p* < 0.05) increase in soluble collagen content in the pig skin. This result can be explained by the disruption of the hydrogen bonds in the collagen triple helix, including intramolecular and intermolecular covalent cross-linking hydrogen bonds, caused by papain and Na_2_CO_3_. Renand et al. [51] and Gerhardy et al. [52] stated that the changes in the collagen content of meat during tenderization were mainly caused by alterations in soluble collagen, and the tenderness of meat showed a strong correlation with the soluble collagen content. Combining these findings with our experimental results on the shear force and TPA, a decrease in soluble collagen content can affect the tenderness of collagen-based meat. The total collagen content of the pork skin decreased continuously at the treatment period of 30–120 min. The 30–90 min treatment decreased the total collagen content of the pork skin to a lesser extent than at the 90–120 min treatment stage. This result may be attributed to the intensified effect of the calcium ion concentration on the calcium-activated enzyme activity during the 90–120 min treatment stage, which resulted in an increase in papain activity; this enabled it to cut the polypeptide chains of collagen [53]. The molecular weight of collagen can be reduced and the solubility of the molecule can be increased through the addition of appropriate amounts of papain. Na_2_CO_3_ regulates the pH of pork skin, which provides it with an alkaline environment. Under alkaline conditions, the acidic soluble collagen in pork skin is released. Xu et al. [17] showed that, for fresh collagen-based meat, high TPA parameters resulted in high total collagen content in the tissue. Our results also support this view.

Slatter et al. [54] revealed that the degree of maturation cross-linking of collagen has an important influence on its solubility. The greater the degree of cross-linking, the lower the collagen solubility, which in turn is one of the factors affecting meat tenderness. Thus, the degree of cross-linking of the collagen in meat directly affects its tenderness. Regarding salt–enzyme–alkali tenderization, we can conclude that the tenderization treatment significantly increased the solubility of the collagen in the pork skin (*p* < 0.05), especially in the Na_2_CO_3_ treatment stage. The increase in collagen solubility reduced the toughness of the meat product, and the mechanical properties of the insoluble collagen fraction determined the meat toughness [55]. No significant change in collagen solubility was observed at 30 min compared with that at 0 min, probably because CaCl_2_ tenderized the pork skin through its ionic strength and calcium-specific effects (possibly proteolytic hydrolysis), which did not primarily involve collagen solubilization. At 60 min, papain disrupted the cross-linked portion of collagen-terminal peptides, which intensified the collagen’s solubilization [56]. During the reaction phase of Na_2_CO_3_, its alkalinity led to the hydrolysis reaction of some peptide bonds of collagen and increased ionic strength in the solution. In addition, the effect of ions may intensify the dissociation and solubilization of collagen molecules. The 120 min treatment group showed a more prominent increase in the rate of collagen solubilization than the other time groups. The change in the collagen dissolution rate was positively correlated with the alteration in elasticity during the TPA study. Li et al. [57] also indicated the possible partial role of the collagen solubility in the increase in elasticity.

### 3.6. Fluorescence Spectroscopy of Collagen

**Figure 3:**
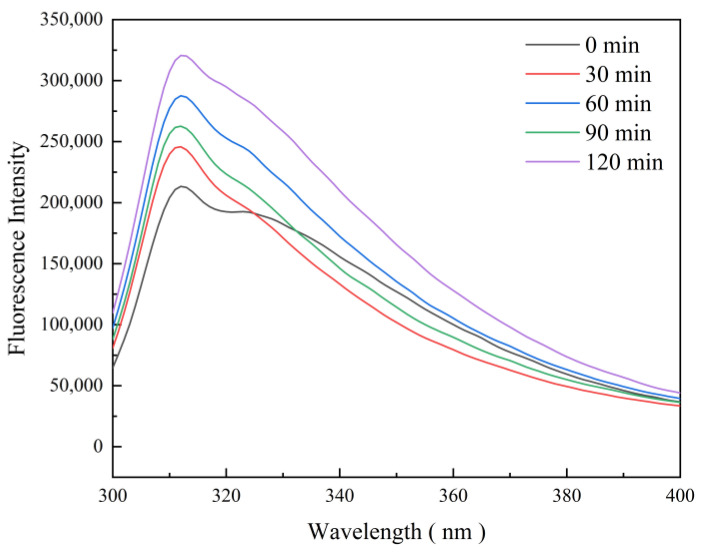
Fluorescence visible spectrum of collagen at different tenderization treatment times.

Figure 3 shows the fluorescence spectra of collagen under salt–enzyme–alkali tenderization treatment with different tenderization times. The endogenous fluorescence intensity of pig skin collagen increased significantly with prolonged tenderization. Thus, the tenderization process can cause an energy transition in collagen, which increases the fluorescence intensity of collagen, exposes a large amount of collagen hydrophobic groups, and alters the tertiary structure of collagen. The maximum emission wavelength of the 30 min treatment group changed from 312.0 nm to 311.4 nm compared with that at 0 min. According to Zhao et al. [58], chloride may disrupt the triple-helical structure of collagen, which leads to the unfolding of its polypeptide chains and the exposure of hydrophobic groups. Therefore, CaCl_2_ favored the unfolding of the molecular structure of pig skin collagen, which exposes the internal tyrosine groups; this condition led to an increase in the intensity of endogenous fluorescence. The maximum emission wavelength of the 60 min treatment group was blue-shifted to 310.8 nm, which suggests that certain portions of the molecular structure of collagen were further exposed, which led to the easier jumping of fluorescent substances between the excited and ground states. Therefore, the emission wavelength shifted in the short-wave direction. The papain treatment degraded the alanine- and hydroxyproline-rich regions of collagen and loosened its structure, which exposed large amounts of tryptophan, causing an increase in the intensity of endogenous fluorescence or the maximum wavelength of emission. In addition, the maximum wavelength of emission in the 90 min treatment group was red-shifted to 313.2 nm. In the alkaline environment of Na_2_CO_3_, the collagen was denatured, and its original tightly packed structure was relaxed and unraveled. Some aromatic amino acids were exposed, which increased the polarity of the collagen solution for easier interaction with polar solvents, such as water. The maximum emission wavelength was red-shifted to 314.3 nm at 120 min, which indicates that the salt–enzyme–alkali rejuvenation treatment can change the collagen structure, increase its solubility, and expose more aromatic amino acid residues, and that papain was able to cleave collagen more efficiently during the treatment to produce smaller peptide fragments.

### 3.7. Infrared Absorption Spectroscopy of Collagen

**Figure 4:**
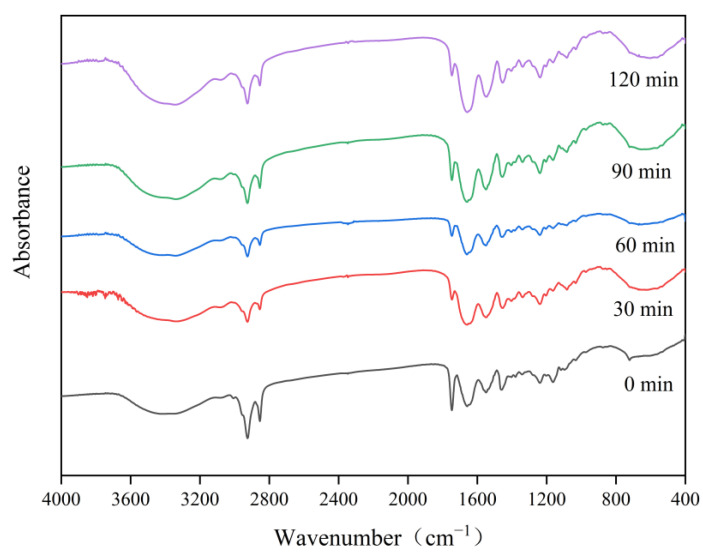
FTIR of collagen at different tenderization treatment times.

FTIR was performed to evaluate the structural changes and degradation of collagen during the combined salt–enzyme–alkali tenderization treatment of pork skin, and the results are shown in Figure 4 and Table 2. The amide A band shows a relation to the stretching vibration of the N-H bond. When the N-H group in a peptide chain contributes to hydrogen bonding, this energy band shifts to a lower frequency [59]. The maximum absorption wavelength of the amide A band of the 30 min treatment group shifted to 3334.97 cm^−1^, in the direction of low wavelength numbers. Clare et al. [60] indicated that the calcium ions in the calcium chloride solution may form a ligand bond with the nitrogen atoms in the amide moiety, and chloride ions may influence the electron density of the amide moiety through ionic–polarizing interactions. These effects may lead to alterations in the structural or vibrational properties of the amide moiety. The maximum absorption wavelengths of the amide A band at 60, 90, and 120 min shifted toward higher wavelengths. This result implies the disruption of some of the hydrogen bonds associated with N-H in collagen during pork skin tenderization. The specific degradation of collagen by papain weakened the hydrogen bonds within the collagen molecules of the pig skin and caused the breakage of some amino acid chains. Na_2_CO_3_ treatment may cause the alkaline modulation of the collagen molecular structure, which affects the interactions between its amino acid residues and causes the deconjugation of part of the collagen or peptide chain breakage [61]. Therefore, the combined tenderization treatment will affect the number of hydrogen bonds within the collagen of porcine skin, which will alter the hydrogen bonding interactions between collagen molecules.

The amide B band is associated with the asymmetric stretching vibration of CH_2_ [62]. The maximum absorption wavelengths of the amide B region in the 30, 60, 90, and 120 min treatment groups shifted toward higher wavelengths compared with those observed at 0 min, namely 2926.27, 2924.84, 2925.32, and 2927.36 cm^−1^, and appeared at higher wavelengths in the 120 min treatment group. This result demonstrates that the combined tenderization treatment altered the hydrophobic interactions within the pig skin collagen.

The amide I band represents the vibrational stretching of the amide carbonyl group (C=O) along the polypeptide backbone, and it shows an association with the differences in the strength of hydrogen bonding associated with the carbonyl group. When C=O participates in relatively strong hydrogen bonding, the position of the amide I band shifts to a lower frequency [61]. The amide I band of the treated pig skin shifted to a lower wave number. This finding implies the production of stronger hydrogen bonds associated with C=O in collagen during the combined tenderization treatment. CaCl_2_, papain, and Na_2_CO_3_ caused a decrease in the structural ordering of the pig skin collagen [12]. However, the 120 min treatment group presented weaker changes at the frequency of the amide I band compared with the 30, 60, and 90 min treatment groups. This result suggests that the combined treatment of pork skins with these three tenderizers can reduce the formation of additional hydrogen bonds associated with C=O during joint tenderization.

Changes in the peak positions of the amide II region reflect alterations in the C-N and N-H bonds in the subunit collagen structure [30]. The amide II region of the 0, 30, 60, 90, and 120 min treatment groups appeared at 1550.63, 1550.21, 1549.21, 1548.98, and 1549.20 cm^−1^, respectively. The typical wave numbers in the amide III region ranged from about 1220 cm^−1^ to 1240 cm^−1^ and exhibited an association with C-N stretching and N-H plane bending vibrations of the amide bond; vibrations of the CH2 group of the glycine backbone and the proline side chain were also included [63]. The intensity of the absorption peaks in the amide III region was reduced at all other times compared with those at 0 min. The maximum absorption wavelength of the amide III region shifted to a greater extent toward lower wavelengths in the 90 and 120 min treatment groups compared with that in the 30 and 60 min treatment groups. This finding indicates the stronger effect of Na_2_CO_3_ on the structure of pig skin collagen than CaCl_2_ and papain. Xiang et al. [64] pointed out that the pH is related to induction and can modify the secondary structure of collagen. Na_2_CO_3_ increased the environmental pH. The pig skin collagen then unfolded, and its internal ordered structure was reduced.

### 3.8. Surface Hydrophobicity Analysis of Collagen

**Figure 5:**
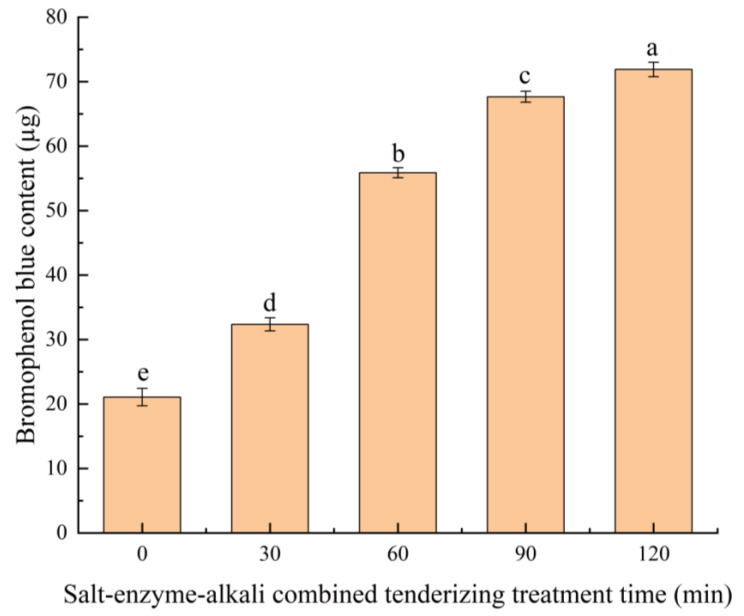
Changes in surface hydrophobicity of collagen during salt–enzyme–alkali combined tenderization treatment. Different letters (a–e) indicate significant differences between different samples (*p* < 0.05).

Bromophenol blue can interact with hydrophobic amino acid residues on the protein surface and reflects the hydrophobic strength and structural changes in proteins [65]. The results are shown in Figure 5. The addition of CaCl_2_ caused a rise in the hydrophobicity of the collagen surface from 0 min to 30 min, which was due to the increased ionic strength of the solution and resulted in the removal of water molecules around the hydrophobic groups on the surfaces of protein molecules to solubilize salt ions, expose hydrophobic groups, and improve the surface hydrophobicity [66]. In the 30–60 min treatment stage, papain cleaved the long peptide chains in the collagen into shorter peptide chains or amino acids, which resulted in the disruption of the original three-dimensional structure, exposing or altering the surface properties of certain hydrophobic regions and improving the surface hydrophobicity. Alizadeh-Pasdar et al. [67] observed that residues with dissociable groups are usually located on the surface of the protein molecule and are therefore directly affected by solutions. A higher rate of increase in the hydrophobicity of the collagen surface was observed in the 60–90 min treatment phase than in the 90–120 min treatment phase. This result was attributed to the Na_2_CO_3_ changing the solution pH, which in turn altered the ionization state of the dissociable groups. This condition led to changes in the protein structure and affected the distribution of hydrophobic amino acid residues within and outside the molecule, which correspondingly altered the hydrophobicity of the collagen surface. During the salt–enzyme–alkali tenderization of pig skin, the tertiary structure of the pig skin collagen reached a dynamic equilibrium; the interaction forces between the collagen molecules, such as disulfide bonding, hydrogen bonding, and van der Waals forces, were impaired; and the collagen structure was constantly stretched, curled, contracted, and broken. Moreover, the hydrophobic groups were gradually exposed, and the surface hydrophobicity of the collagen increased. However, when the amount of pig skin collagen decomposed by Na_2_CO_3_ was extremely large, the broken collagen molecules coalesced to cover the hydrophobic groups, and the surface hydrophobicity of the collagen decreased.

## 4. Conclusions

This study aimed to investigate the effect of salt–enzyme–alkali staged progressive tenderization treatments on the texture of pork skins and the mechanisms behind it. The quality characteristics of the pig skin were considerably influenced by the salt, enzyme, and alkali tenderization treatments, which could lead to texture softening. The texture of the pork skin was related to the soluble and insoluble collagen components produced by collagen fiber degradation. The CaCl_2_ treatment had a negligible effect on all quantitative collagen types (total, soluble, or insoluble collagen) in the pork skin during tenderization. Papain cleaved the polypeptide chains of collagen, and Na_2_CO_3_ raised the ambient pH. Both events resulted in a substantial decrease in insoluble collagen components and an increase in soluble collagen components containing low-molecular-weight polypeptides. The decrease in insoluble collagen led to the rejuvenation of the connective tissue. In addition, the initial treatment of CaCl_2_ fully activated the calcium-activated enzymes and hindered the activity of the calcium-activated enzyme inhibitors through the addition of exogenous calcium ions. As a result, papain attained a high level of collagen decomposition efficiency and thus contributed to the tenderization of the pork rinds and an improvement in water retention in the meat. The Na_2_CO_3_ treatment not only enhanced the enzyme activity but also effectively neutralized CaCl_2_ and papain and their by-products, which further enhanced the tenderizing effect. In addition, the increased pH during the salt–enzyme–alkali tenderization process could alter the morphology of the pork rinds from well-organized stacks with a good texture to loose filamentous structures. Meanwhile, the Fourier infrared spectra, fluorescence spectra, and surface hydrophobicity of collagen observed during tenderization proved that the salt–enzyme–alkali tenderization treatment could break collagen’s hydrogen bonds, expose hydrophobic groups, and impair the disulfide bonds, hydrogen bonds, and van der Waals forces, which contributed to changes in the tertiary structure of the collagen. An increase in the fluorescence intensity implied an increase in collagen destruction, which reduced the chewiness and hardness of the pork rinds and improved their tenderness. The increase in surface hydrophobicity is negatively correlated with the hardness and chewiness of pork rinds, and it improves the tenderness of the meat, thus increasing the market acceptance of ready-to-eat pork rinds. The solubilization of soluble collagen and the alteration of the collagen structure contributed to the process of pork skin tenderization. In summary, we investigated the changes in the texture, structure, and function of pig skin after salt–enzyme–alkali treatment, and the results provide theoretical support and guidance for the application of pig skin collagen in the fields of food, medicine, and biology. However, only 1-year-old pig skin was used as the experimental material in this study; if the age of the animal is different, the initial structure of the collagen (intermolecular cross-linking, etc.) will also be different. This will affect the results of any rejuvenation treatments. Future studies could consider pig skin of different ages to more fully assess the effect of age on the collagen properties and rejuvenation effects. Attention could also be given to consumer preferences and market research to more fully assess the impact of different treatments on consumers. Meanwhile, the salt–enzyme–alkali tenderization treatment significantly improved the texture and collagen properties of the pork skins, providing a new technological option for the food industry. In order to maximize the application of this technology, we recommend that the optimal treatment conditions are used when treating different types of pork skin, depending on their thickness and origin. In addition, this technology is equally applicable to other animal by-products, such as cowhides and sheepskins, and good results can be achieved by appropriately adjusting the treatment conditions. Therefore, we encourage researchers and enterprises in the industry to actively explore these potential applications to further enhance the added value and market competitiveness of animal by-products.

## Figures and Tables

**Table 1:** Proximate composition, shear, pH, color, and TPA of pork skin during salt–enzyme–alkali tenderization treatment.

	0 min	30 min	60 min	90 min	120 min
Moisture content (g/100 g)	54.17 ± 0.92 ^d^	60.56 ± 0.94 ^c^	62.32 ± 0.55 ^b^	64.06 ± 1.04 ^a^	63.30 ± 0.36 ^a^
Protein content (g/100 g)	24.69 ± 0.33 ^a^	23.77 ± 0.22 ^b^	21.95 ± 0.36 ^c^	19.76 ± 0.20 ^d^	18.46 ± 0.44 ^e^
Fat content (g/100 g)	23.12 ± 0.70 ^a^	21.81 ± 0.92 ^b^	20.82 ± 0.17 ^c^	17.29 ± 0.01 ^d^	15.90 ± 0.17 ^e^
Shear force (N)	31.39 ± 0.71 ^a^	28.23 ± 0.69 ^b^	20.91 ± 0.57 ^c^	17.25 ± 0.37 ^d^	12.09 ± 0.47 ^e^
Hardness (N)	12,640.65 ± 792.90 ^a^	11,765.25 ± 379.53 ^a^	9761.85 ± 1063.93 ^b^	7440.19 ± 795.01 ^c^	5396.62 ± 775.45 ^d^
Masticatory (MJ)	9432.14 ± 172.75 ^a^	8615.92 ± 238.43 ^b^	6680.65 ± 135.05 ^c^	5757.68 ± 133.11 ^d^	4557.05 ± 88.58 ^e^
Elasticity (MM)	0.8 ± 0.05 ^d^	0.92 ± 0.03 ^d^	1.16 ± 0.05 ^c^	1.35 ± 0.06 ^b^	1.78 ± 0.07 ^a^

Note: Different letters in the same line (^a–e^) indicate significant differences between different samples (*p* < 0.05).

**Table 2:** Peak position of amide band in collagen treated by different tenderizing processes.

Salt–Enzyme–Alkali Combined Tenderizing Treatment Time (min)	Amide A Wave Number (cm^−1^)	Amide B Wave Number (cm^−1^)	Amide Ⅰ Wave Number (cm^−1^)	Amide Ⅱ Wave Number (cm^−1^)	Amide Ⅲ Wave Number (cm^−1^)
0	3336.94	2923.42	1659.86	1550.63	1241.05
30	3334.97	2926.27	1658.44	1550.21	1240.92
60	3337.82	2924.84	1657.28	1549.21	1239.77
90	3340.66	2925.32	1654.47	1548.98	1238.24
120	3341.09	2927.36	1658.54	1549.20	1237.34

## Data Availability

The original contributions presented in the study are included in the article, further inquiries can be directed to the corresponding author.
